# Pteropine orthoreoviruses use cell surface heparan sulphate as an attachment receptor

**DOI:** 10.1080/22221751.2023.2208683

**Published:** 2023-05-16

**Authors:** Chee Wah Tan, Akshamal M. Gamage, Wee Chee Yap, Leon Jia Wei Tang, Yuan Sun, Xing-Lou Yang, Alyssa Pyke, Kaw Bing Chua, Lin-Fa Wang

**Affiliations:** a Programme in Emerging Infectious Diseases, Duke-NUS Medical School, Singapore, Singapore; b Integrative Sciences and Engineering Programme, National University of Singapore, Singapore, Singapore; c College of Animal Science, South China Agricultural University, Guangzhou, People’s Republic of China; d Chinese Academy of Sciences, Kunming Institute of Zoology, Kunming, People’s Republic of China; e Department of Health, Public Health Virology Laboratory, Forensic and Scientific Services, Queensland Government, Coopers Plains, Australia; f Temasek Lifesciences Laboratory, National University of Singapore, Singapore

**Keywords:** Pteropine orthoreovirus, heparan sulphate, attachment receptor, Melaka virus, glycosaminoglycans

## Abstract

Pteropine orthoreoviruses (PRVs) are an emerging group of fusogenic, bat-borne viruses from the *Orthoreovirus* genus. Since the isolation of PRV from a patient with acute respiratory tract infections in 2006, the zoonotic potential of PRV has been further highlighted following subsequent isolation of PRV species from patients in Malaysia, Hong Kong and Indonesia. However, the entry mechanism of PRV is currently unknown. In this study, we investigated the role of previously identified mammalian orthoreovirus (MRV) receptors, sialic acid and junctional adhesion molecule-1 for PRV infection. However, none of these receptors played a significant role in PRV infection, suggesting PRV uses a distinct entry receptor from MRV. Given its broad tissue tropism, we hypothesized that PRV may use a receptor that is widely expressed in all cell types, heparan sulphate (HS). Enzymatic removal of cell surface HS by heparinase treatment and genetic ablation of HS biosynthesis genes, SLC35B2, exostosin-1, N-deacetylase/N-sulfotransferase I and beta-1,3-glucuronyltransferase 3, significantly reduced infection with multiple genetically distinct PRV species. Replication kinetic of PRV3M in HS knockout cells revealed that HS plays a crucial role in the early phase of PRV infection. Mechanistic studies demonstrated that HS is an essential host-factor for PRV attachment and internalization into cells. To our knowledge, this is the first report on the use of HS as an attachment receptor by PRVs.

## Introduction

Pteropine orthoreoviruses (PRVs) are non-enveloped, double-stranded RNA viruses classified within the *Orthoreovirus* genus in the *Reoviridae* family [1]. The *Reoviridae* family is composed of 15 genera and includes several common human pathogens, particularly within the rotaviruses and orthoreoviruses [2]. The first documented PRV species, PRV1N (alternatively known as Nelson Bay virus), was isolated in 1968 from a grey-headed flying fox (*Pteropus policephalus*) in Nelson Bay, Australia [1]. In 1999, a second PRV species (PRV2P, also known as Pulau virus) was isolated from pooled urine samples from *Pteropus hypomelanus* in Tioman Island, Malaysia [3]. The first bat-to-human transmission of PRV was reported in 2006, in which a bat flew through the door into the 39-years old patient’s living hall and flew “frantically” for about 2–3 min before fly out. The patient developed fever with severe respiratory diseases a week post-exposure to the unusual event. A fusogenic orthoreovirus closely related to Nelson Bay virus was isolated from the patient. The virus was named after the place of isolation, known as Melaka virus (alternatively known as PRV3M) [4]. Since then, multiple PRVs were isolated from human patients and bats from Southeast Asia [5–16]. A recent study by Tee et al. [17] reported that out of 632 urban outpatients who tested negative for all known respiratory viruses, 2.2% were positive against PRV. Serological studies on selected populations in South East Asia revealed PRV seroprevalence rates ranging from 4.4% to 13% [12,18]. In a recent study by our group, cynomolgus macaques were demonstrated to act as a potential reservoir or intermediate host for PRV [19].

Reovirus virions are composed of two concentric capsid shells. The inner capsid encapsulates the 10 dsRNA genome segments and contains enzymes needed to launch virus replication upon entry into cells [2]. The outer capsid contains three capsid proteins, σ1, σ3 and µ1, that play important roles in cell attachment and entry [20]. The σ3 and µ1 proteins form 200 heterohexamers that decorate the outer capsid [2,21]. Attachment of the virion to the host cell occurs via trimers of the σ1 protein [20,22,23]. All serotypes of mammalian orthoreoviruses (MRV) engage the proteinaceous receptor junctional adhesion molecule-1 (JAM-1) as an attachment receptor [22,24]. MRV serotype 1 and 3 also bind GM2 and sialic acid apart from JAM-1, respectively [25,26]. Once attached to the cell surface, β1 integrin and Ngr1 facilitate MRV entry into host cells [27,28]. However, despite 50 years after the first isolation of PRV and increasing evidence of its zoonotic transmission capability, the cellular receptor for PRV remains uncharacterized.

Receptor binding is an essential event during viral infection. The ability to recognize and interact with specific receptors determines the host range and tissue tropism [29]. The broad tissue tropism of PRV implies the use of host receptors that are widely expressed across cell types [30]. Heparan sulphate proteoglycans (HSPGs) are unbranched, negatively charged heparan sulphate (HS) polysaccharides attached to a variety of cell surface or extracellular matrix proteins. HSPGs are ubiquitously expressed in most cell types and mediate various biological activities [31]. Many viruses exploit HS as attachment factor to increase their docking efficiency with specific entry receptors [32]. For example, herpes simplex virus-1 (HSV-1) uses 3-O-HS as an entry receptor [33].

In this study, we explore the role of two MRV attachment receptors, sialic acid and JAM-1, in PRV infection. However, none of these receptors were observed to play a significant role in PRV infection. We further investigate the role of HS in PRV infection. Using a combination of CRISPR-Cas9 knockout cells and enzymatic assays, we show that HS plays an important role in PRV infection. Mechanistic studies revealed that cell surface HS support PRV attachment and internalization into host cells.

## Results

### Known MRV receptors have no significant role in PRV3M infection

**Figure 1.**
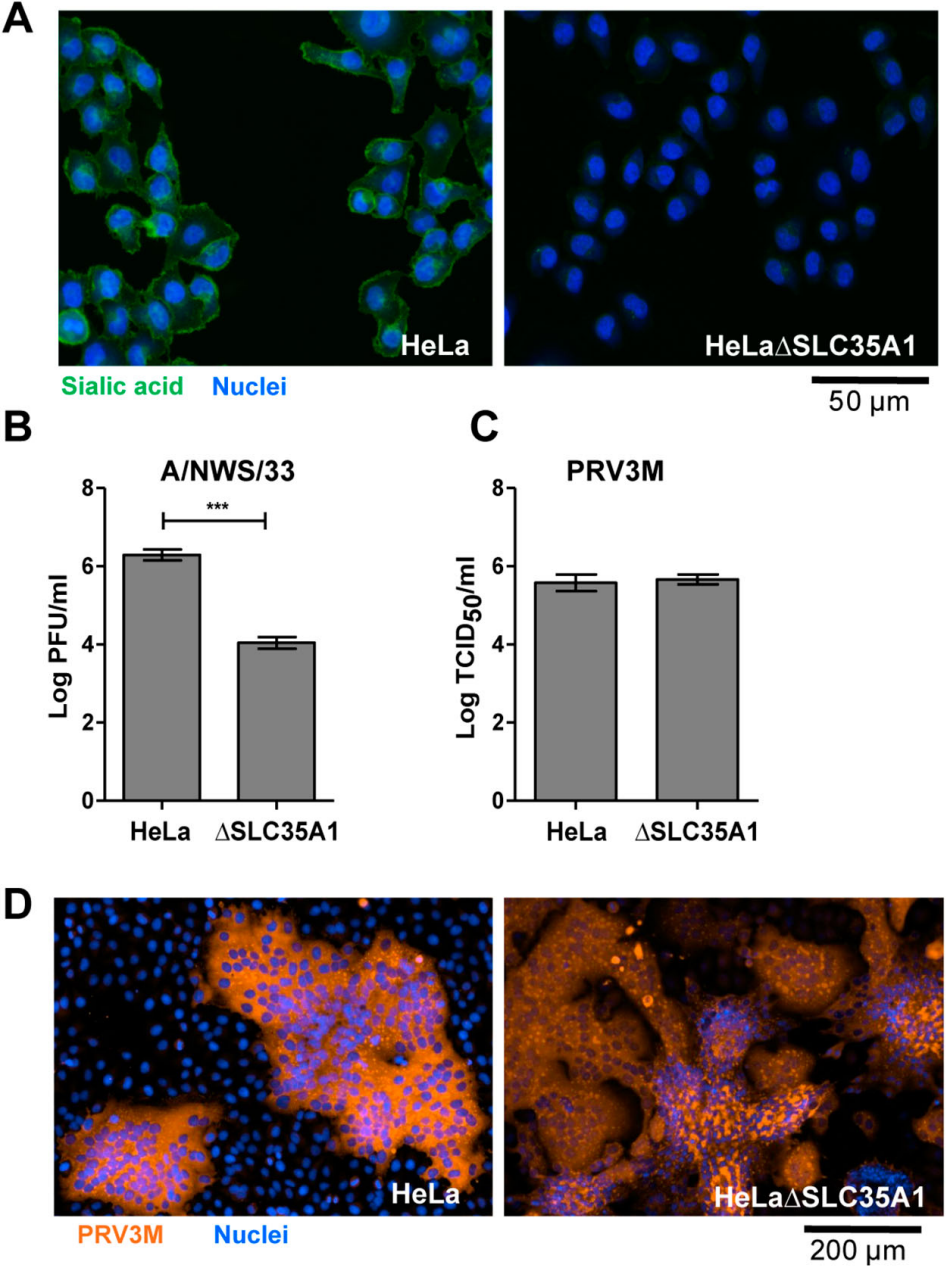
Genetic ablation of cell surface sialic acid expression and its impact on PRV infection. (A) HeLa and HeLaΔSLC35A1 cells were immunostained with FITC-conjugated WGA. Green fluorescence indicates sialic acid expression. Susceptibility of HeLa and HeLaΔSLC35A1 cells to (B) influenza A/NWS/33 and (C) PRV3M infections. Data presented were virus yield at 48 hpi. (D) PRV-induced syncytial formation was observed via immunofluorescent staining for viral antigen. Blue indicates cell nuclei and brown indicates PRV3M antigens. All experiments were repeated for at least two biological replicates. Asterisks indicate statistically significant differences (*P < 0.05; **P < 0.01; ***P < 0.001). Error represent means ± standard error.

Sialic acids serve as the attachment receptor for MRV type 3 [23,25]. Sialic acid knockout HeLa cells were constructed via CRISPR-Cas9 targeting of Solute Carrier Family 35 (Uridine 5′-diphosphate (UDP) Galactose transporter), member A1 (SLC35A1). SLC35A1 is found in the Golgi apparatus membrane, where it transports Cytidine monophosphate (CMP)-sialic acid from the cytosol into the Golgi for glycosylation. SLC35A1 is therefore required for sialylation on the cell surface. HeLaΔSLC35A1 cells were deficient in sialic acid, as validated by FITC-conjugated wheat germ agglutinin (WGA) lectin staining (Figure 1(A)). HeLaΔSLC35A1 cells were resistant to influenza A/NWS/33 infection, a virus previously demonstrated to utilize sialic acid as an attachment and entry receptor (Figure 1(B)). However, sialic acid-deficient HeLa cells remained susceptible to PRV3M infection, as sialic-acid-deficient cells did not have a difference in viral titres after PRV3M infection (Figure 1(C)) nor any difference in immunofluorescence staining for virus antigen (Figure 1(D)) compared to wild-type cells. This implied that sialic acid has no role in PRV infection. Cells deficient in JAM-1, an MRV attachment receptor, were also susceptible to PRV3M infection, but significantly abolished MRV-1 and MRV-2 infection (Supplementary Figure 1(A–C)). Neuraminidase-treated JAM-1 knockout cells lacked cell surface sialylation (Supplementary Figure 1(B)) and had no impact on PRV3M infection, but further reduced MRV-2 infection (Supplementary Figure 1(C)). Sequence analysis suggested that the PRVs µ1 outer capsid protein does not contain integrin β1 binding motifs, Arginine-Glycine-Aspartic acid and Lysine-Glycine-Glutamic acid (data not shown), implying integrin β1 was also unlikely to serve as an entry receptor for PRV. Taken together, these data demonstrate that PRV uses a distinct cellular receptor to those previously reported for MRV.

### HS facilitates PRV infection

**Figure 2.**
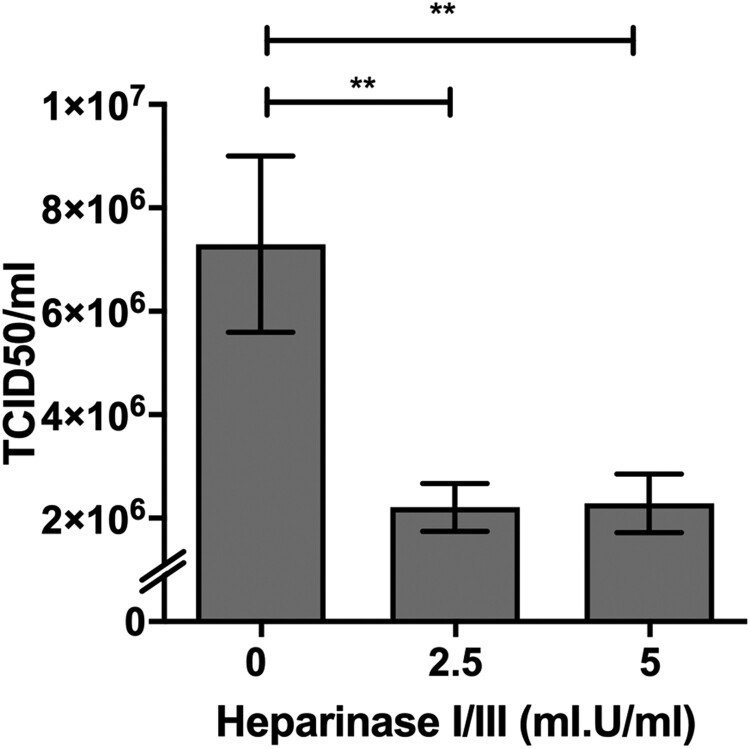
Impact of heparinases I and III treatment on PRV infection of HeLa cells. HeLa cells were pre-treated with heparinase I and III blend (Sigma) for 1 h prior PRV3M infection. Total virus yields were determined at 48 hpi by end-point dilution. Experiments were repeated for at least two biological replicates. Asterisks indicate statistically significant differences (*P < 0.05; **P < 0.01; ***P < 0.001). Error represent means ± standard error.

**Figure 3.**
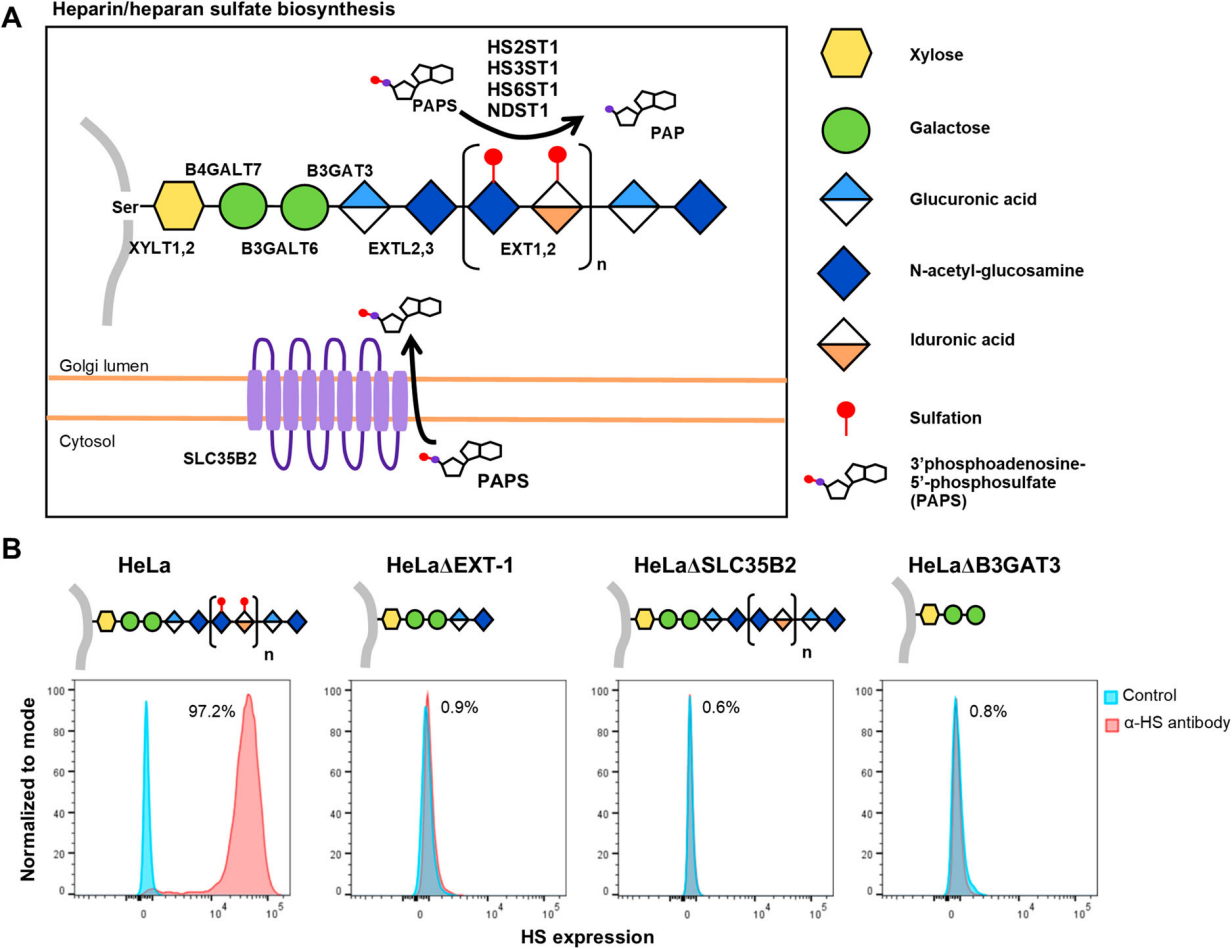
Illustration of HS biosynthesis pathway, and effect of genetic ablation of HS biosynthesis genes on HS expression. (A) HS biosynthesis takes place in the Golgi lumen. Xylosyltransferases (XYLT1,2), galactosltransferases (B4GALT7, 6) and glucuronyltransferase (B3GAT3) involved in the biosynthesis of xylose-galactose-galactose-glucuronic acid protein-glycan linkage. N-acetylglucosaminyltransferase (EXTL2) catalyze synthesis of the glucuronic acid-N-acetyl-glucosamine. EXT1,2 catalyze chain elongation with N-acetyl-glucosamine and glucuronic acid. Glucuronyl C5-epimerase performs epimerization of glucuronic acid to l-iduronic acid and adenosine 3′-phospho 5′-phosphosulphate (PAPS) transporter 1 (SLC35B2) transport the PAPS from cytosol into Golgi lumen. N-deacetylase/N-sulfotranferases (NDST1–4) perform deacetylation and N-sulphation of the N-acetyl-glucosamine. HS 2-O-sulfotransferase is involved in transferring of sulphate group to the 2′ of the iduronic acid residue of HS. Both HS 3-O and HS 6-O sulfotransferases are involved in transferring of sulphate group to the 3′ and 6′ of the N-acetyl-glucosamine residue of the HS. (B) Illustration of HS biosynthesis in ΔEXT-1, ΔSLC35B2 and ΔB3GAT3 cells. HS expression in HeLa wild-type and knockout cells were analysed via flow cytometry using anti-HS monoclonal antibody, F58-10E4. All experiments were repeated for at least two biological replicates.

PRV species are known to exhibit broad cell-type and species tropism [4]. HS is ubiquitously expressed across different cell types and are a known attachment and entry factor for multiple viruses. We speculated that PRV could use HS as an attachment factor. Heparinase treatment of HeLa cells significantly impaired PRV infection (Figure 2). Next, multiple HS knockout cells were generated by targeting exostosin-1 (EXT-1), beta-1,3-glucuronyltransferase 3 (B3GAT3) and adenosine 3′-phospho 5′-phosphosulphate transporter 1 (SLC35B2), genes involved in different steps in HS biosynthesis (Figure 3(A)). EXT-1 encodes an endoplasmic reticulum-resident type II transmembrane glycosyltransferase involved in the chain elongation step of HS biosynthesis. Cells deficient in EXT-1 are impaired in HS biosynthesis [34]. B3GAT3 encodes a glucuronyltransferase which catalyzes glycosaminoglycan-protein linkage via a glucuronyl transfer reaction. B3GAT3 knockout cells are deficient in HS, chondroitin sulphate (CS) and dermatan sulphate (DS) synthesis [34]. SLC35B2 is located in the microsomal membrane and transport 3′-phosphoadenosine 5′-phosphosulphate (PAPS) from the cytosol into Golgi lumen for sulphation of glycoproteins, proteoglycans and glycolipids (Figure 3(A)). HeLa cells with CRISPR-Cas9 mediated knockout of each of these genes were demonstrated to lack HS expression via flow cytometry analysis using an anti-HS antibody (Figure 3(B)).

**Figure 4.**
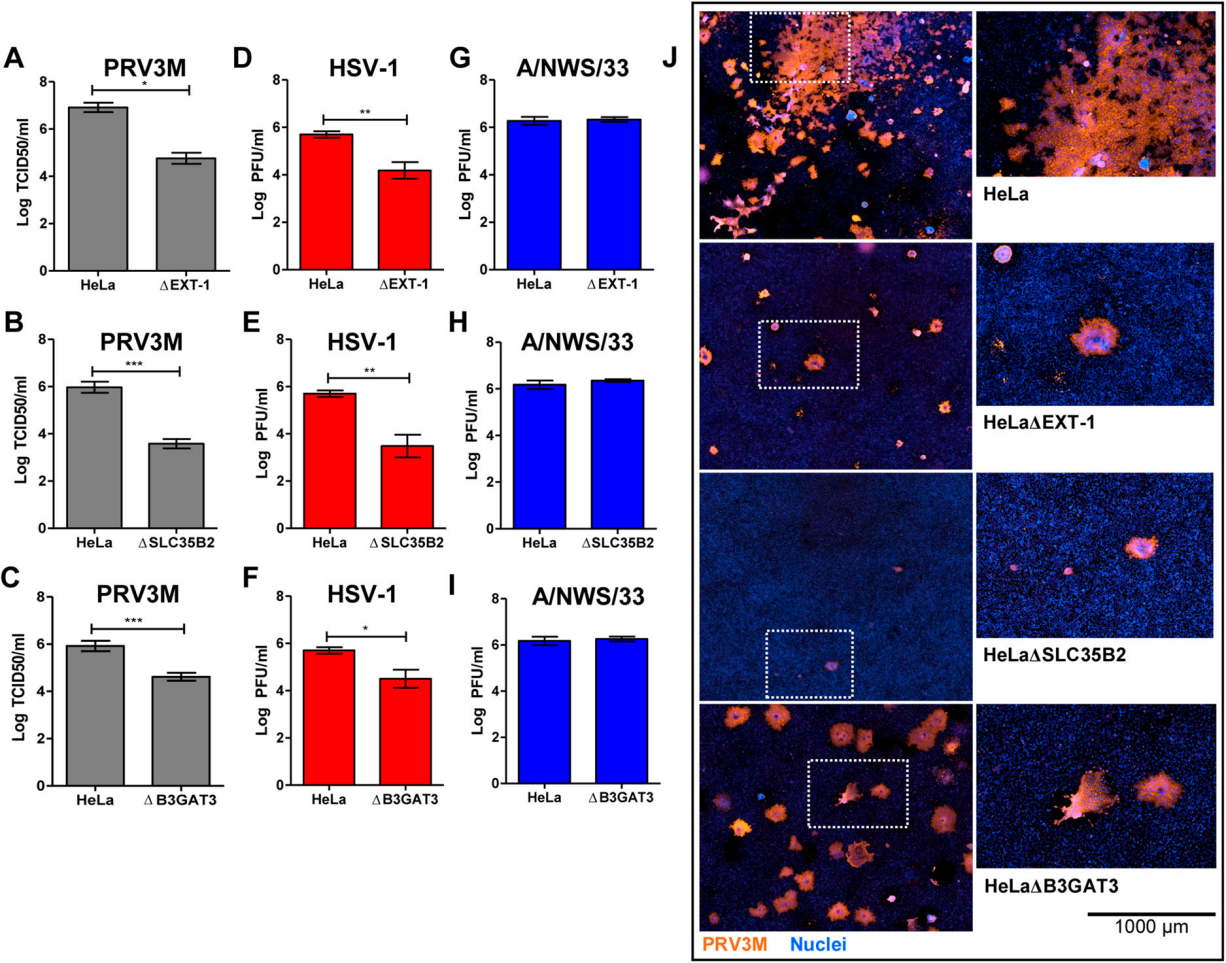
Effect of HS deficiency in PRV3M infection. The susceptibility of HeLaΔEXT-1, HeLaΔSLC35B2 and HeLaΔB3GAT3 cells to (A–C) PRV3M, (D–F) HSV-1 and (G–I) influenza A/NWS/33. The data presented were virus yields at 48 hpi. (J) Immunofluorescent staining for viral antigen was performed to validate PRV3M infection in HeLa, HeLaΔEXT-1, HeLaΔSLC35B2 and HeLaΔB3GAT3 cells. Blue indicates cell nuclei and brown indicates PRV3M antigens. Scale bar is for the enlarged images. All experiments were repeated for at least two biological replicates. Asterisks indicate statistically significant differences (*P < 0.05; **P < 0.01; ***P < 0.001). Error represent means ± standard error.

As demonstrated in Figure 4(A–C), HeLaΔEXT-1, HeLaΔSLC35B2 and HeLaΔB3GAT3 were less susceptible to PRV3M infection, with up to 2.2 log TCID50/ml, 2.4 log TCID50/ml and 1.3 log TCID50/ml reduction, respectively, compared to wild-type cells. Cells deficient in HS were also observed to be less susceptible to HSV-1 (Figure 4(D–F)), a virus known to use HS as an attachment factor. In addition, genetic ablation of HS biosynthesis had no impact on influenza A/NWS/33 infection, a virus species which has not been reported to utilize HS for attachment or entry (Figure 4(G–I)). Immunofluorescence staining for virus antigen confirmed that HeLaΔEXT-1 and HeLaΔSLC35B2 are less susceptible to PRV3M infection (Figure 4(J)). Taken together, these data demonstrate that the sulphation present on HS are critical for PRV infection. The role of HS in PRV infection is not restricted to HeLa cells, but also in a neuroblastoma SH-SY5Y cells (Supplementary Figure 2). Notably, HeLa cells are deficient in both HS and CS; HeLaΔB3GAT3 have less impact on PRV3M infection as compared to HeLaΔEXT-1 and HeLaΔSLC35B2 (Figure 4(C,J)). This suggests that CS could act as decoy to prevent PRV3M infection.

### HS facilitates multiple genetically diverse PRV infection

**Figure 5.**
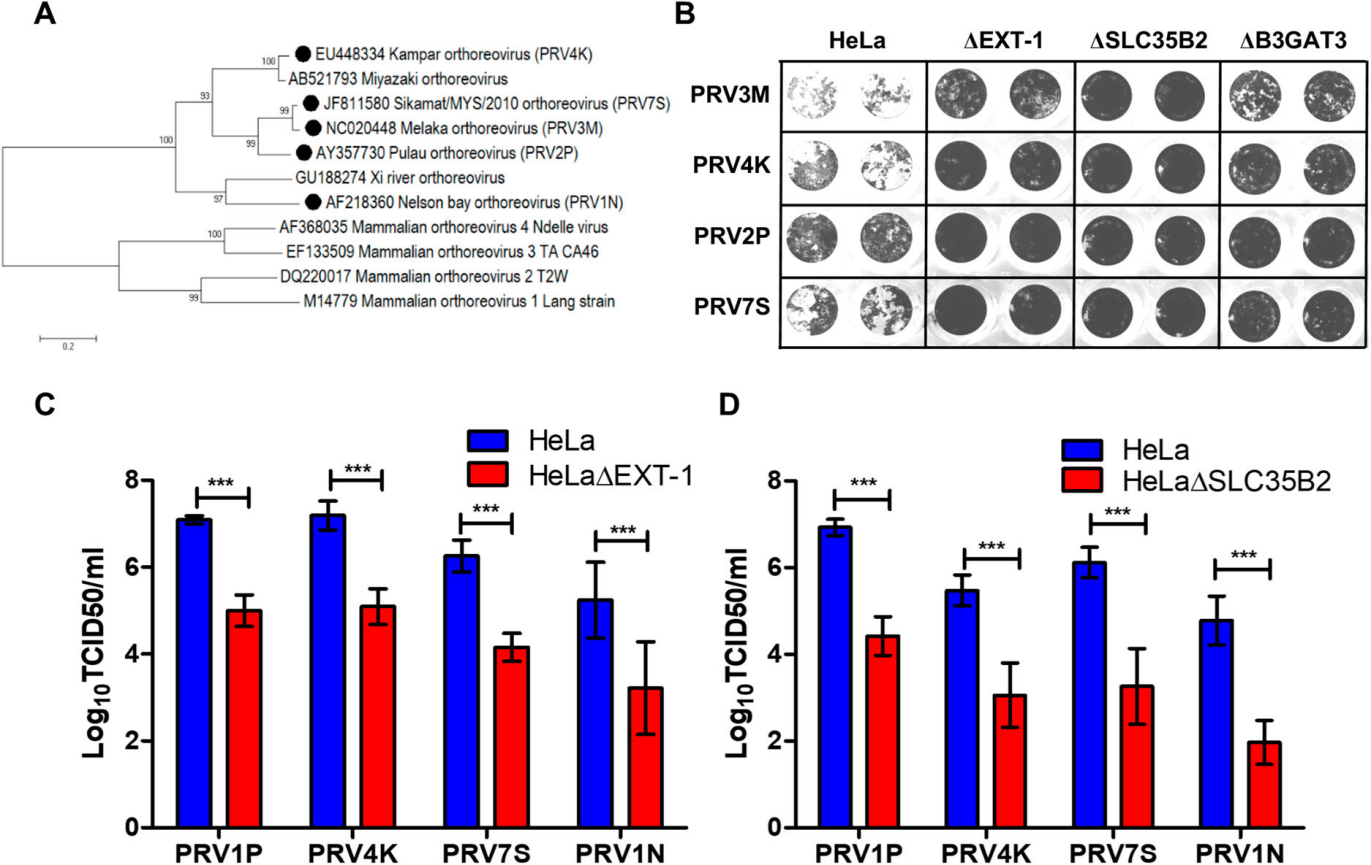
Role of HS in facilitating infection by multiple genetically diverse PRV species. (A) Phylogenetic analysis of PRV and MRV S1 genes. Phylogenetic tree was built using neighbour joining method with 1000 bootstrapping. Filled circles indicate the PRV species used in this study. (B) The syncytia formation of PRV2P, PRV3M, PRV4K and PRV7S infected HeLa, HeLaΔEXT-1, HeLaΔSLC35B2 and HeLaΔB3GAT3. At 48 hpi, the cells were fixed with 4% paraformaldehyde and stained with 0.2% crystal violet. Susceptibility of (C) HeLaΔEXT-1 and (D) HeLaΔSLC35B2 to PRV1N, PRV2P, PRV4K and PRV7S infection. The virus titres were determined at 48 hpi by end-point dilution. All experiments were repeated for at least two biological replicates. Asterisks indicate statistically significant differences (*P < 0.05; **P < 0.01; ***P < 0.001). Error represent means ± standard error.

Next, we characterized the role of HS in multiple genetically diverse PRV species, including PRV1N, PRV2P, PRV4K and PRV7S (Figure 5(A)). Both PRV1N and PRV2P are isolated from bats, while the remaining species are isolated from patients with respiratory illness. Upon infection with all four PRV species, HeLaΔEXT-1, HeLaΔSLC35B2 and HeLaΔB3GAT3 displayed less syncytial formation as compared to wild-type HeLa (Figure 5(B)). HeLaΔEXT-1 cells displayed significantly reduced PRV2P, PRV4K, PRV7S and PRV1N titres, with approximately 2 log TCID50/ml reduction compared to wild-type cells (Figure 5(C)). HeLaΔSLC35B2 exhibited the least susceptibility to PRV infection, with about 2.4–2.9 log TCID50/ml reduction across different PRV strains (Figure 5(D)). Our data demonstrate that there was no significant difference in HS usage between PRV strains and show that HS binding ability is conserved across PRV species.

### Reconstitution of HS biosynthesis restores PRV susceptibility

**Figure 6.**
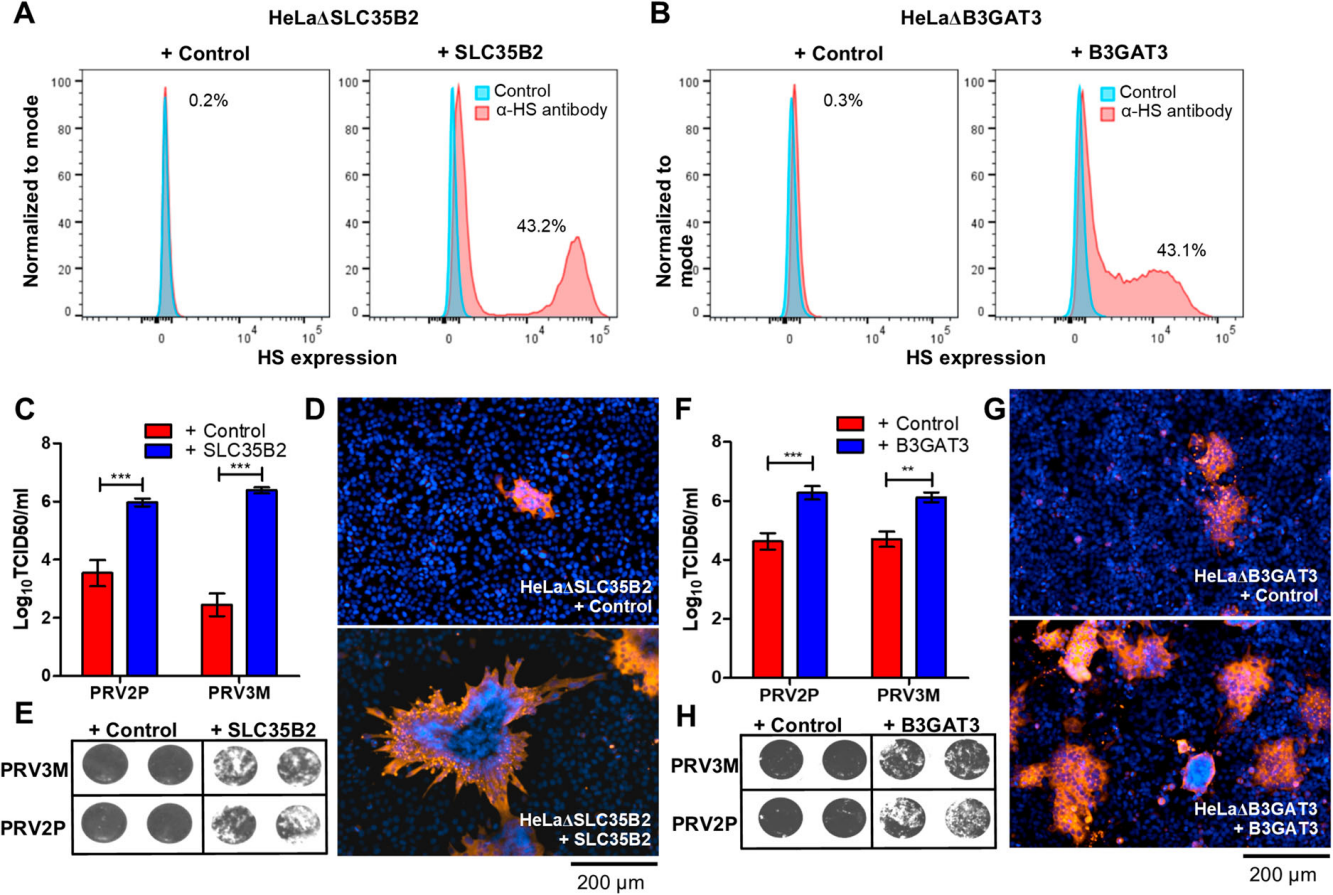
Reconstitution of knockout genes restores HS biosynthesis and PRV3M infectivity. (A, B) Flow cytometry analysis of HeLaΔSLC35B2 and HeLaΔB3GAT3 transduced with lentiviruses for stable expression of SLC35B2 and B3GAT3, respectively. Knockout cells transduced with an empty vector were used as control. Effect of HeLaΔSLC35B2 transduced with lentivirus expressing SLC35B2 and empty vector on PRV3M and PRV2P infection, as measured via (C) virus yields, (D) immunofluorescence staining for viral antigen and (E) virus-induced syncytial formation. B3GAT3 or empty vector transduced HeLaΔB3GAT3 were infected with PRV3M and PRV2P at a MOI of 1. The effect on (F) virus yields, (G) immunofluorescence staining for viral antigen and (H) syncytial formation of PRV2P and PRV3M were determined. All experiments were repeated for at least two biological replicates. Asterisks indicate statistically significant differences (*P < 0.05; **P < 0.01; ***P < 0.001). Error represent means ± standard error.

Next, we reconstituted HS expression by transducing knockout cells with lentiviruses carrying the respective genes in each of the knockout cells. Reconstitution of SLC35B2 and B3GAT3 in HeLaΔSLC35B2 and HeLaΔB3GAT3, respectively, recovered HS expression on the cell surface (Figure 6(A,B)). Reconstituted SLC35B2 in HeLaΔSLC35B2 cells regained susceptibility to PRV3M and PRV2P infection, with 3.9 log TCID50/ml and 2.4 log TCID50/ml increase in viral titres, respectively (Figure 6(C)). The results were further validated by immunofluorescence assay for virus antigen, and syncytial formation upon infection (Figure 6(D,E)). Restoration of B3GAT3 expression in HeLaΔB3GAT3 restored PRV3M and PRV2P infection, as validated by virus yields, immunofluorescence staining and virus-induced syncytial formation (Figure 6(F–H)).

### N-linked sulphation is critical for PRV infection

**Figure 7.**
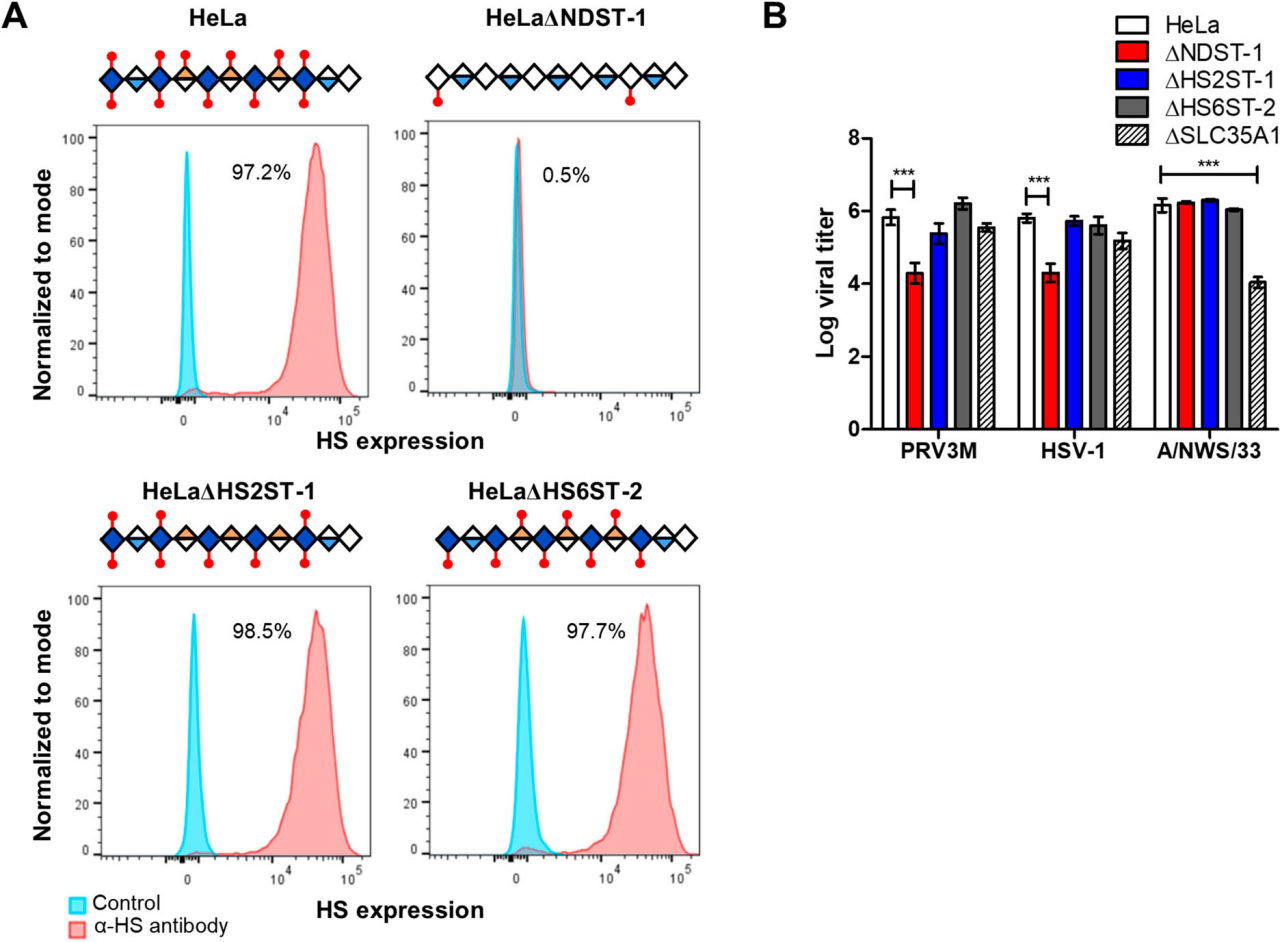
Effect of genetic ablation of genes involved in HS modifications on susceptibility to PRV3M infection. (A) Graphical illustration of HS chain in NDST-1, HS2ST-1 and HS6ST-2 deficiency. Blue diamond indicates de-acetyl-glucosamine; open diamond indicates N-acetyl-glucosamine, half blue diamond indicates glucuronic acid; half orange diamond indicates iduronic acid and red mark indicates sulphation. Flow cytometry histogram plots of HeLa, HeLaΔNDST-1, HeLaΔHS2ST-1 and HeLaΔHS6ST-2 cells on surface HS expression. (B) Effect of NDST-1, HS2ST-1 and HS6ST-2 knockout in PRV3M, HSV-1 and A/NWS/33 infection. All experiments were repeated for at least two biological replicates. Asterisks indicate statistically significant differences (*P < 0.05; **P < 0.01; ***P < 0.001). Bar charts represent mean ± standard error.

To further characterize the type of sulphation critical for PRV binding, we generated individual gene-knockouts for N-deacetylase/N-sulfotransferase I (NDST-1), HS 2-O-sulfotransferase-1 (HS2ST-1) and HS 6-O-sulfotransferase-2 (HS6ST-2). NDST-1 is a type II transmembrane protein that catalyzes the transfer of sulphation from PAPS to form an N-linked sulphation on glucosamine in HS. Both HS2ST-1 and HS6ST-2 are involved in transferring a sulphate group to the 2′ and 6′ of the iduronic acid residue of HS, respectively [34]. All knockout cells were validated for homozygous indel formation by genomic DNA sequencing. HeLaΔNDST-1 cells showed loss of anti-HS antibody clone F58-10E4 binding, while the antibody was still capable of recognizing HS epitopes present on the HeLaΔHS2ST-1 and HeLaΔHS6ST-2 cells (Figure 7(A)). HeLa cells deficient in N-linked sulphation, but not O-linked sulphation significantly abolished PRV3M infection, with 1.5 log TCID50/ml reduction in HeLaΔNDST-1 (Figure 7(B)). These data indicate that N-linked sulphation on HS is critical for PRV infection, while O-linked sulphation-deficient cells likely have sufficient sulphation on the HS to facilitate viral infection.

## HS-deficient cells impact PRV attachment to host cells

**Figure 8.**
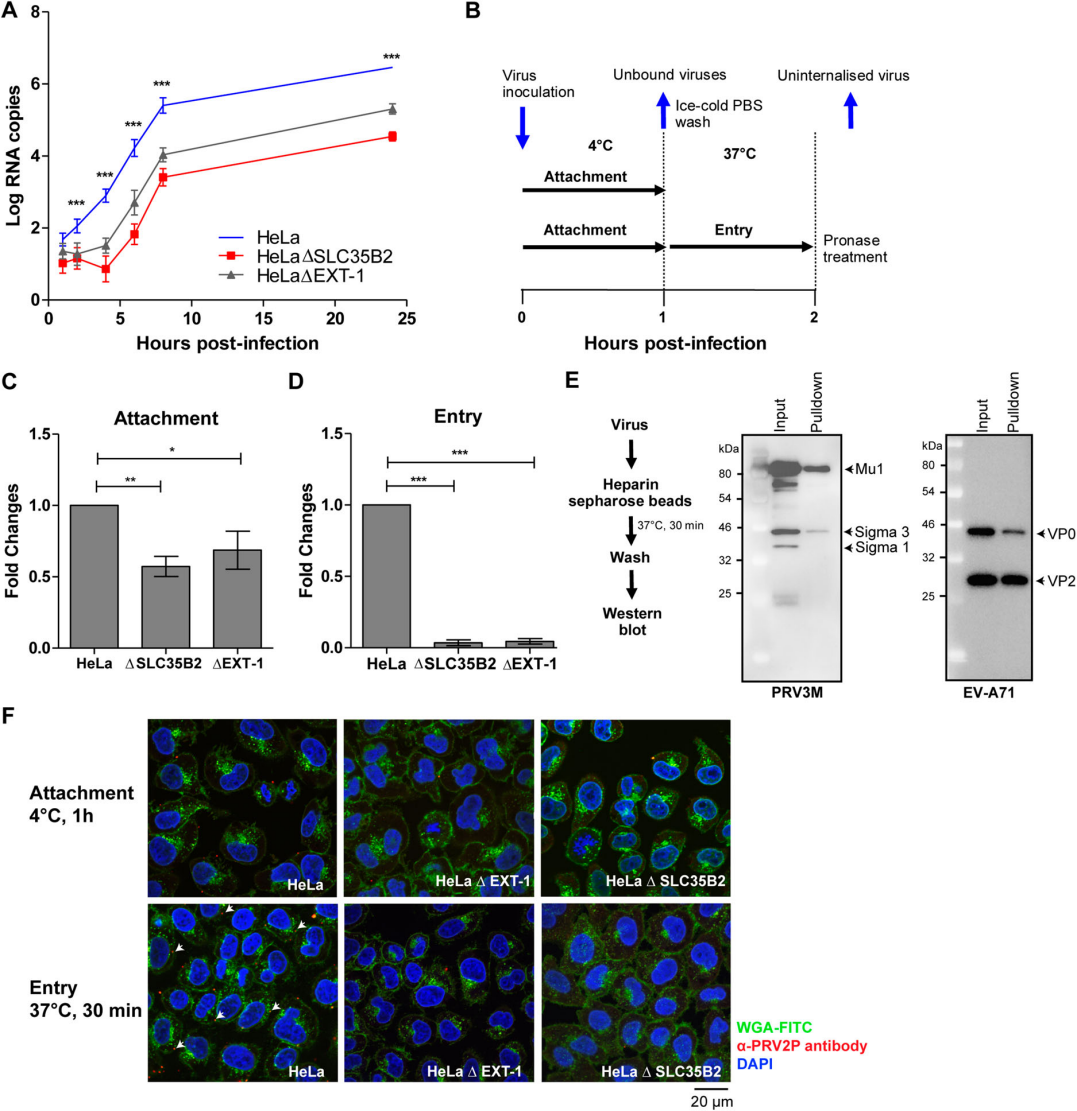
Replication kinetics and mechanistic studies of PRV3M attachment and entry. (A) Replication kinetics of PRV3M in HeLa, HeLaΔEXT-1 and HeLaΔSLC35B2 cells. Viral RNA copies at 1, 2, 4, 6, 8, 24 hpi were quantified by real-time PCR. (B) Illustration of experimental procedure to characterize virus attachment and entry. Ice-cold PRV3M inoculum was added on to pre-chilled HeLa, HeLaΔEXT-1 and HeLaΔSLC35B2 cells and incubated on ice for 1 h. At 1 hpi, cells were washed three times with ice-cold DPBS to remove unbound viruses. Infected cells were either harvested for total RNA extraction or incubated at 37°C incubation for 1 h to promote virus entry. Cells were then incubated with pronase at a final concentration of 1 mg/ml for 10 min at 37°C. This step removes membrane-bound, but uninternalized virus particles. Pronase-treated cells were subjected to extensive washes prior to total RNA extraction. (C) Effect of EXT-1 and SLC35B2 knockout on PRV3M attachment. (D) Attachment of PRV3M and EV-A71 virion to heparin sepharose beads followed by Western blot analysis. (E) Internalization analysis of PRV3M in EXT-1 and SLC35B2 knockout cells. Fold changes of viral gRNA were determined using 2^−ΔΔCT^ method after normalization with a housekeeping gene, SNRPD3. (F) Confocal analysis of PRV3M attachment and entry. Cell membrane (green) was immunostained with FITC-WGA, PRV3M (red) was immunostained with antiserum derived from rabbit hyperimmunized with PRV2P and nuclei (blue) were stained with DAPI. Arrow indicates internalized viral particles. The images were acquired using Nikon N-STORM confocal microscope at 60× magnification. All experiments were repeated for at least two biological replicates. Asterisks indicate statistically significant differences (*P < 0.05; **P < 0.01; ***P < 0.001). Error represent means ± standard error.

Lastly, PRV3M replication kinetics were assayed in HeLa, HeLaΔSLC35B2 and HeLaΔEXT-1 cells. PRV3M replication in HeLaΔSLC35B2 and HeLaΔEXT-1 was significantly reduced at early infection time-points (2–6 hpi), compared to infection in wild-type HeLa cells (Figure 8(A)). This indicated that HS deficiency abolished PRV3M attachment or entry into host cells. To further characterize the mechanism underlying HS-dependent PRV infection, we allowed PRV3M attachment to the cell surface at 4°C, followed by 37°C to promote viral entry. Uninternalized membrane-bound viruses were removed by pronase treatment (Figure 8(B)). HS-deficient cells displayed significantly reduced PRV virion attachment at 4°C (Figure 8(C)). When the temperature was shifted to 37°C to promote entry, 99.97% and 99.96% of the PRV3M particles remained uninternalized in HeLaΔSLC35B2 and HeLaΔEXT-1, respectively (Figure 8(D)). To further validate the HS binding ability, we performed a pull-down experiment using heparin sepharose [35–38]. Heparin, found mainly in mast cells, is a highly sulphated form of polydisperse linear polysaccharide which displays higher N- and O-sulphation than HS [39]. PRV3M virion was added into heparin sepharose beads followed by Western blot analysis. PRV3M virion were observed to directly interact with the heparin sepharose beads (Figure 8(E)), supporting the role of HS as a PRV attachment receptor. Enterovirus A71 was used as a heparin binding positive control (Figure 8(E)). Using confocal microscopy analysis, we observed that cells deficient in HS synthesis or sulphation had significantly reduced PRV3M attachment and internalization into the cells (Figure 8(F)). Overall, our data support a role for HS as the attachment receptor during PRV infection.

## Discussion

PRV species are fusogenic orthoreoviruses that are pathogenic and cause various severe symptoms in vertebrates. Multiple studies have reported PRV as causative agents of severe respiratory tract infections in humans, suggesting that infections by bat-borne orthoreoviruses may represent potential zoonotic threats [4–6,12,15]. Virus–host receptor interaction is essential to establish successful infection. To date, the virus–host interaction of PRV is under-studied and the functional entry receptor of PRV remains unidentified. In this study, we investigated the role of known MRV receptors in PRV infection. The C-terminal globular head domain of MRV σ1 plays a key role in JAM-1 interaction [24]. Our JAM-1 knockout HeLa cells failed to reduce PRV infection, consistent with observations by Kawagishi et al., that the σC protein of PRV1N does not bind to JAM-1 expressing L929 cells [40]. Apart from JAM-1, type 3 MRV σ1 protein has been reported to bind α2,3-linked and α2,6-linked sialic acid [25]. Our previous study revealed that sialic acid-deficient VeroΔGNE cells and α2,3-linked sialic acid-deficient Huh7ΔST3GAL4 cells failed to reduce PRV3M infection [41]. In this study, we further investigate the role of sialic acid in HeLa cells by knocking out the CMP-sialic acid transporter, SLC35A1. Similar to our previous finding, HeLaΔSLC35A1 remained susceptible to PRV3M infection. Our findings demonstrate that both JAM-1 and sialic acid have no role in PRV attachment or entry. Interestingly, sialic acid-deficient cells enhanced virus-induced syncytial formation, implying more efficient virus entry into host cells. Bechara et al. [42] previously demonstrated that internalization of the HS binding peptide was more efficient in sialic acid-deficient cells, as the sialic acid may sequester and prevent peptides from interacting with HS. Therefore, this observation in sialic acid-deficient cells is further consistent with a central role for HS during PRV infection.

HS proteoglycans are glycoproteins carrying one or more covalently bound HS chains, a large anionic polysaccharide of the glycosaminoglycan family. HSPGs have been found to bind a wide range of protein ligands, including cytokines, chemokines and growth factors [31]. Because of its abundance on the cell surface, several viral pathogens utilize HSPG as attachment factor prior to interacting with the entry receptor [32]. This includes HSV-1 which binds HS prior to interacting with entry receptors [33,43,44], enterovirus A71 (EV-A71) which binds HS prior to interacting with scavenger receptor class B2 [35,45], HIV-1 which binds HS prior to interact with the CXCR4 co-receptor [46], and also human papillomavirus (HPV) [47] and adenoviruses [48]. HSV-1 is known to bind HS followed by nectin-1/2 and herpesvirus entry mediators to promote viral entry. In cells deficient for these entry factors, 3-O sulphated HS is sufficient to mediate HSV-1 attachment and fusion. Thus far, HSV-1 is the only virus reported to use HS as an entry receptor [33].

In the current study, we have demonstrated that cell surface HS plays a critical role in PRV infection, both by enzymatic removal of HS and genetic ablation of HS biosynthesis genes. We constructed HeLa cells with only HS-deficient (ΔEXT-1); HS, CS and DS-deficient (ΔB3GAT3) and sulphation-deficient (ΔSLC35B2) proteoglycans. Cells with SLC35B2 knockout exhibited the least susceptibility to PRV infection, suggesting that other sulphated glycoproteins and glycolipids may play a role in PRV infection. However, cells deficient in major glycosaminoglycans have the least impact on PRV susceptibility, suggesting that CS and DS could act as decoy receptors and protect cells from PRV infection. During HS biosynthesis, the HS chains undergo various modifications, including N-deacetylation/N-sulphation of N-acetyl-glucosamine, epimerization of d-glucuronic acid to l-iduronic acid, sulphation at the 2-O position of l-iduronic acid and sulphation at 3 and 6-O position of N-acetyl-glucosamine [31,34]. Through genetic ablation of genes involved in HS modifications, we identified that N-sulphation of N-acetyl-glucosamine plays a critical role in PRV infection. Surprisingly, further mechanistic work revealed that PRV uses HS as an attachment receptor and internalization factor. HS-deficient cells do not completely abolish PRV infections, implying the existence of alternative entry pathway(s).

Based on these results, we propose that PRV binds to HS proteoglycan through cell attachment protein (σ1) and major outer capsid protein (σ3) (Supplementary Figure 3). HS proteoglycan could act as endocytic receptors and undergo constitutive as well as ligand-induced endocytosis [49]. The precise mechanism of endocytosis is unclear. A recent study by Kawaguchi et al. [50] identified the HS proteoglycan as a receptor for clathrin-mediated endocytosis of HS binding arginine-rich cell penetrating peptides. We therefore postulate that PRV utilizes a similar mechanism for host cell entry.

Multiple studies have reported that HS usage by viruses could result as a tissue culture adaptation during virus propagation [32,37,51–56]. The usage of early passage virus stocks and consistent observations across different PRV species minimizes this possibility. The HS binding domain of PRV remains unknown. Screening of the three outer capsid proteins, σ1, σ3 and µ1, for putative arginine/lysine rich domains was unsuccessful, suggesting that the HS binding domains could be conformation dependent. In summary, we have identified that PRV uses cell surface HS as an attachment receptor. Genetic ablation of HS biosynthesis genes almost completely abolished PRV infection, implying HS is a major attachment receptor for PRV. To our knowledge, this is the first documented instance of a pteropine orthoreovirus utilizing HS for host cell invasion.

## Materials and methods

### Cells and viruses

Human cervical carcinoma (HeLa, ATCC # CCL-2) cells, human embryonic kidney (HEK293, ATCC # CRL-1573) cells, human neuroblastoma (SH-SY5Y, ATCC # CRL-2266) cells, African green monkey kidney (Vero, ATCC # CCL-81) cells, rhabdomyosarcoma (RD; ATCC # CCL-136) cells were grown and maintained in Dulbecco’s modified Eagle medium (DMEM) supplemented with 10% foetal bovine serum (FBS; Hyclone), 50 units/ml of penicillin and 50 units/ml of streptomycin (Gibco).

PRV1N (commonly known as Nelson Bay virus), PRV2P (also known as Pulau virus), PRV3M (also known as Melaka virus), PRV4K (commonly known as Kampar virus) and PRV7S (known as Sikamat virus) were propagated in Vero cells. PRV1N and PRV2P were isolated from bats from Nelson Bay, Australia and Tioman Island, Malaysia, respectively [1,3]. PRV3M, PRV4K and PRV7S were isolated from patients with severe respiratory illness from Melaka, Kampar and Sikamat, Malaysia, respectively [4–6]. MRVs type 1 and 2 were propagated in Madin-Darby canine kidney (MDCK) cells. Human influenza virus A/NWS/33 (ATCC # VR-219) was propagated in MDCK cells in DMEM supplemented with 0.3% bovine serum albumin (BSA), 25 mM HEPES and 1 µg/ml L-1-tosylamido-2-phenylethyl chloromethyl ketone (TPCK) -treated trypsin. HSV-1 was propagated in Vero cells. EV-A71 strain 5845/SIN/000009 was propagated in RD cells. The virus titres of PRV and MRV were determined by end-point dilution, TCID50 using Vero cells. The virus titres of influenza A/NWS/33, EV-A71 and HSV-1 were determined by plaque assay using MDCK, RD and Vero cells, respectively. HeLa cells were highly susceptible to PRV infection and, therefore, were used to characterize the role of HS in PRV infection.

### Gene editing using CRISPR-Cas9

The EXT-1, SLC35B2, SLC35A1, B3GAT3, HS6ST1, HS2ST1, NDST-1 and JAM-1 sgRNA oligos were synthesized and cloned into lentiCRISPRv2 (#52961, Addgene) as previously described [57]. The lentiCRISPRv2 plasmid were then co-transfected to HEK293T cells with pMDLg/pRRE (#12253, Addgene), pRSV-Rev (#12251, Addgene) and pMD2.G (#12259, Addgene). At 48 post-transfections, the supernatant containing lentiviruses were collected and clarified by low-speed centrifugation. To generate CRISPR knockout cells, monolayer HeLa or SH-SY5Y cells were transduced with respective lentiviruses at a multiplicity of infection (MOI) of 0.3. At 48 hpt, 2 µg/ml of puromycin was added and clonal expansion was performed at 5 days post-antibiotic selection. All knockout clones were verified by DNA sequencing and flow cytometry analysis.

### Enzymatic removal of cell surface HS

HeLa cells at 2 × 10^4^ cells density were pre-treated with various concentration of heparinases I/III blend (Sigma, USA) in serum-free DMEM for 1 h at 37°C, followed by three Dulbecco's modified phosphate buffered saline (DPBS) wash. The treated and untreated cells were infected with PRV3M at a MOI of 1 for 1 h at 37°C. After 1 hpi, the inoculum was removed and the unbound viruses were removed by three DPBS washing step. Infected cells were replenished with 2% FBS DMEM and the viruses were harvested at 48 hpi for viral titration.

### Restoration of SLC35B2 and B3GAT3 expression

Total mRNA of HeLa cells was extracted using E.Z.N.A total RNA extraction kit (Omega). cDNA synthesis was performed using ImProm-II reverse transcription system (Promega) according to manufacturer’s instructions. Gene specific primers for respective genes were used to amplify the open reading frame using Q5 high fidelity DNA polymerase (NEB) following manufacturer’s instructions. The polymerase chain reaction (PCR) amplicons were agarose gel-purified, followed by restriction digestion with *Not*I and *Pac*I restriction enzyme (NEB). The *Not*I and *Pac*I digested amplicons were cloned into pQCXIH vector (Clonetech). All clones were verified by DNA sequencing. Lentiviruses were produced by co-transfection of pQCXIH vector which carried the respective genes pMD2.G (#12259, Addgene) into GP2-293 cells (Clonetech) and the lentiviruses were harvested at 48 h post-transfection. To restore the knockout gene, the knockout cells were transduced with the respectively lentivirus at a MOI of 0.5 for 48 h, followed by hygromycin B Gold (InvivoGen) at final concentration of 100 µg/ml. The HS expression of the restored cells was verified by flow cytometry analysis.

## Immunofluorescent analysis of PRV3M infection

PRV3M-infected cells in a 24-well plate were fixed with 4% formaldehyde for 10 min followed by permeabilization with 0.25% Triton-X-100 in phosphate buffer saline (PBS). Cells were then blocked with 1% BSA in DPBS for 1 h at room temperature. PRV3M antigens were immunostained with 1:200 diluted serum of PRV3M-infected macaque or 1:400 antiserum derived from PRV2P immunized rabbit and 1:400 diluted secondary PE-conjugated anti-monkey antibody (Abcam) or 1:400 diluted Phycoerythrin (PE)-labelled anti-rabbit antibody (Invitrogen) for 2 h and 1 h, respectively, at room temperature. Cell nuclei were stained with 4′,6-diamidino-2-phenylindole, dihydrochloride (DAPI) (Sigma) for 10 min. Immunofluorescence was detected and captured using Cytation 5 imager (BioTek).

## Flow cytometry analysis of cell surface HS

HeLa wild-type, knockout and reconstituted cells were seeded at a density of 5 × 10^4^ cells/well in a 24-well tissue culture treated plate overnight. The following day, cells were detached by exposure to 10 mM Ethylenediaminetetraacetic acid (EDTA) for 10 min at 37°C, followed by vigorous pipetting. Cells were pelleted by centrifugation and resuspended in Fluorescence activated cell sorting (FACS) buffer (DPBS with 2% FBS and 5 mM EDTA), stained with primary anti-HS monoclonal antibody, clone F58-10E4 (AMSBIO, UK) at a 1:100 dilution for 45 min at 4°C, followed by PE-conjugated goat-anti-mouse IgM (Biolegend) staining at a 1:200 dilution for 30 min at 4°C. Cells were washed twice in FACS buffer, and flow cytometry acquisition performed on a BD LSRFortessa (BD Biosciences). FlowJo V10 software (Tree Star Inc.) was used for analysis of flow cytometry data.

## Virus attachment and entry assay

HeLa, HeLaΔSLC35B2 and HeLa ΔEXT-1 were infected with PRV3M at a MOI of 1 for 1 h at 4°C. The infected cells were washed three times with DPBS, followed by 37°C incubation for 1 h to promote virus entry. Cells were then treated with pronase (Roche) for 10 min at 37°C to remove uninternalized virus particle. Pronase-treated cells were washed five times with DPBS and the total RNA was extracted using E.Z.N.A Total RNA kit I (Omega BioTek). PRV3M and housekeeping, small nuclear ribonucleoprotein D3 (SNRPD3) mRNA levels were determined by real-time PCR using PRV3M S4-F (5′ CAT TGT CAC TCC GAT TAT GG 3′) and PRV3M S4-R (TGG GAG GGT GCA GAG CAT AG). In brief, cDNA was synthesized using Quantitect Reverse Transcription kit (Qiagen) and real-time PCR was performed with CFX96 real-time PCR detection system (Bio-Rad) using SensiFast SYBR No-ROX kit (Bioline). Fold changes of viral gRNA were determined using 2^−ΔΔCT^ method after normalization with a housekeeping gene, SNRPD3.

For confocal analysis, HeLa, HeLaΔSLC35B2 and HeLa ΔEXT-1 were infected with sucrose-cushion concentrated PRV3M at a MOI of 50 for 1 h at 4°C. The infected cells were washed three times with DPBS, followed by 37°C incubation for 30 min to promote virus entry. Cells were fixed with paraformaldehyde for 30 min followed by permeabilization with 0.1% Triton-X-100 for 10 min. The PRV3M virion were stained with 1:400 diluted antiserum derived from PRV2P hyperimmunized rabbit, followed by 1:400 diluted PE-labelled anti-rabbit IgG (Invitrogen) for 30 min at 37°C. Cell membrane and nuclei were stained with WGA-Fluorescein isothiocyanate (FITC) and DAPI, respectively, for 10 min at room temperature. The fluorescence images were acquired with Nikon N-STORM confocal microscope.

## Heparin binding analysis

Sucrose-cushion concentrated PRV3M and EV-A71 particles were pre-incubated with heparin sepharose (Abcam) for 30 min at 37°C with agitation. The unbound viral particles were removed by five DPBS washes. The sepharose beads were resuspended with the 1× Sodium dodecyl-sulfate polyacrylamide gel electrophoresis (SDS-PAGE) loading dye, subjected to SDS-PAGE and Western blot analysis. The PRV3M and EV-A71 viral proteins were detected using anti-PRV2P rabbit antiserum and Light Diagnostic enterovirus 71/coxsackievirus A16 reagent (3323, Millipore), respectively, followed by horseradish peroxidase (HRP) conjugated secondary detecting antibody.

## Statistical analysis

Statistical details of experiments are found in the corresponding figure legends. The comparison of HS-deficient cells and wild-type cells were analysed by the Student’s *t* test or one-way Analysis of Variance (ANOVA) for multiple comparison. Statistical analyses were conducted with GraphPad Prism 8 (GraphPad). *P* values of <0.05 were considered statistically significant.

## Supplementary Material

Supplemental Material
