# Supplementary material for: Pteropine orthoreoviruses use cell surface heparan sulphate as an attachment receptor
Source: Emerg Microbes Infect. 2023 May 16;12(1):2208683. doi: 10.1080/22221751.2023.2208683 (PMC10190195; doi:10.1080/22221751.2023.2208683)
Supplement: Supplemental Material [file TEMI_A_2208683_SM2651.pdf]

Supplementary Material

Pteropine orthoreoviruses use cell surface heparan sulfate as an attachment receptor

Chee Wah Tan[1,*], Akshamal M. Gamage[1], Wee Chee Yap[1], Leon Jia Wei Tang[1,2], Yuan Sun[1,3], Xing-Lou Yang[1,4], Alyssa Pyke[5], Kaw Bing Chua[6], Lin-Fa Wang[1,*]

[1]Programme in Emerging Infectious Diseases, Duke-NUS Medical School, Singapore.
[2]Integrative Sciences and Engineering Programme, National University of Singapore, Singapore.
[3]College of Animal Science, South China Agricultural University, Guangzhou, China
[4]Kunming Institute of Zoology, Chinese Academy of Sciences, Kunming, China
[5]Department of Health, Public Health Virology Laboratory, Forensic and Scientific Services, Queensland Government, Coopers Plains, Australia.
[6]Temasek Lifesciences Laboratory, Singapore.

Short title: Heparan sulfate facilitates Pteropine orthoreoviruses attachment

Keywords: Pteropine orthoreovirus, Heparan sulfate, attachment receptor

*Corresponding author
Lin-Fa Wang (linfa.wang@duke-nus.edu.sg)
Chee Wah Tan (cheewah.tan@duke-nus.edu.sg)
Programme in Emerging Infectious Diseases,
Duke-NUS Medical School, Singapore.

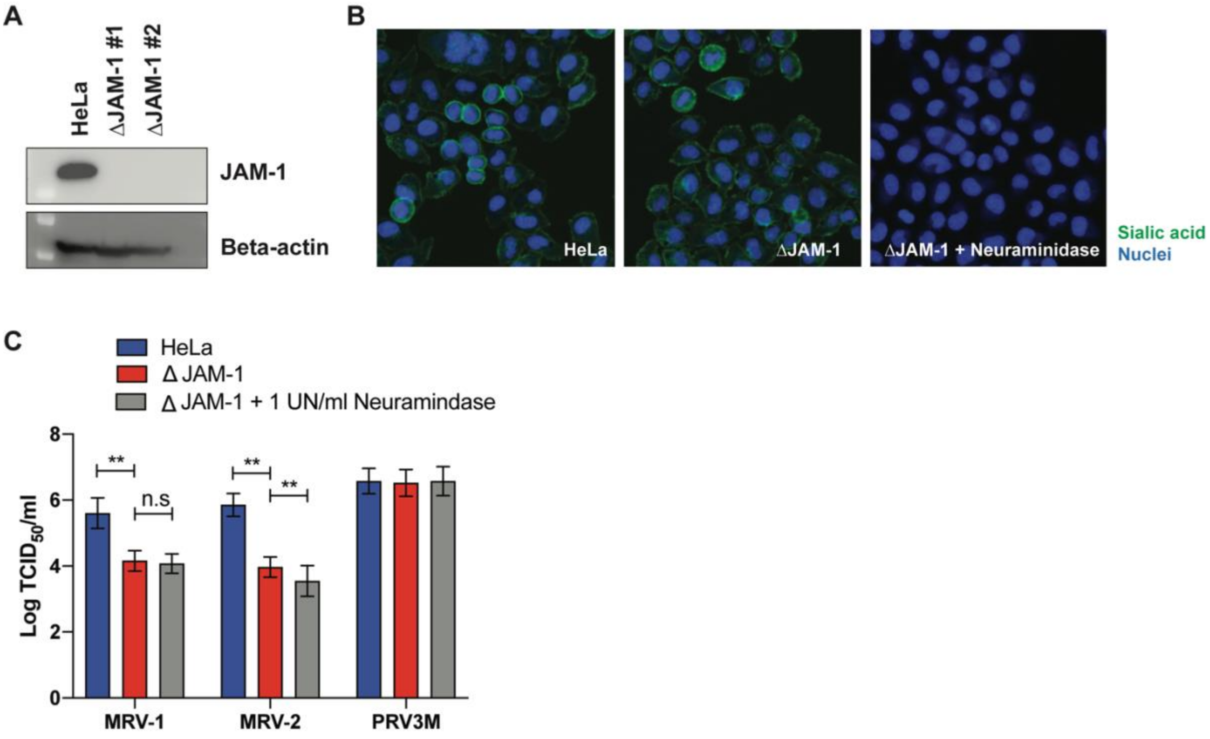

Supplementary Fig 1. Role of junction adhesion molecule-1 (JAM-1) and sialic acid in PRV3M infection. (A) Western blot detection of JAM-1 in HeLa and HeLaΔJAM-1 cells. The JAM-1 was detected using rabbit anti-JAM-1 monoclonal antibody (Abcam, ab52647, 1:5000) and the β-actin was detected using mouse anti-Beta actin monoclonal antibody (Sigma, A2228, 1:10000). (B) Immunostaining and detection of cell surface sialic acid after 1UN/ml neuraminidase *C. perfringens* (Sigma, N2133) treatment. Sialic acid (green) was immunostained with FITC-WGA (2 μg/ml) and the nuclei (blue) were stained with DAPI. (C) Effect of JAM-1 and sialic acid-deficient cells on MRVs and PRV3M infection. The viral titers were determined at 48 hours post-infection and expressed in Log TCID50/ml. All experiments were repeated for three biological replicates. Asterisks indicate statistically significant differences (**$P < 0.01$). Error bars indicate standard deviation.

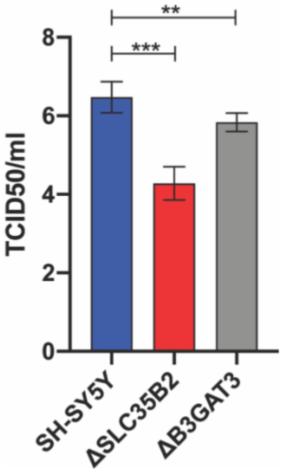

Supplementary Fig 2. Role of HS in PRV3M infection in SH-SY5Y. The susceptibility of SH-SY5Y, SH-SY5YΔSLC35B2, and SH-SY5YΔB3GAT3 cells to PRV3M infection was determined by infecting knockout cells with PRV3M at an MOI of 0.1. The viral titers were determined at 48 hours post-infection and expressed in Log TCID50/ml. All experiments were repeated for three biological replicates. Asterisks indicate statistically significant differences (**P < 0.01; ***P<0.001). Error bars indicate standard deviation.

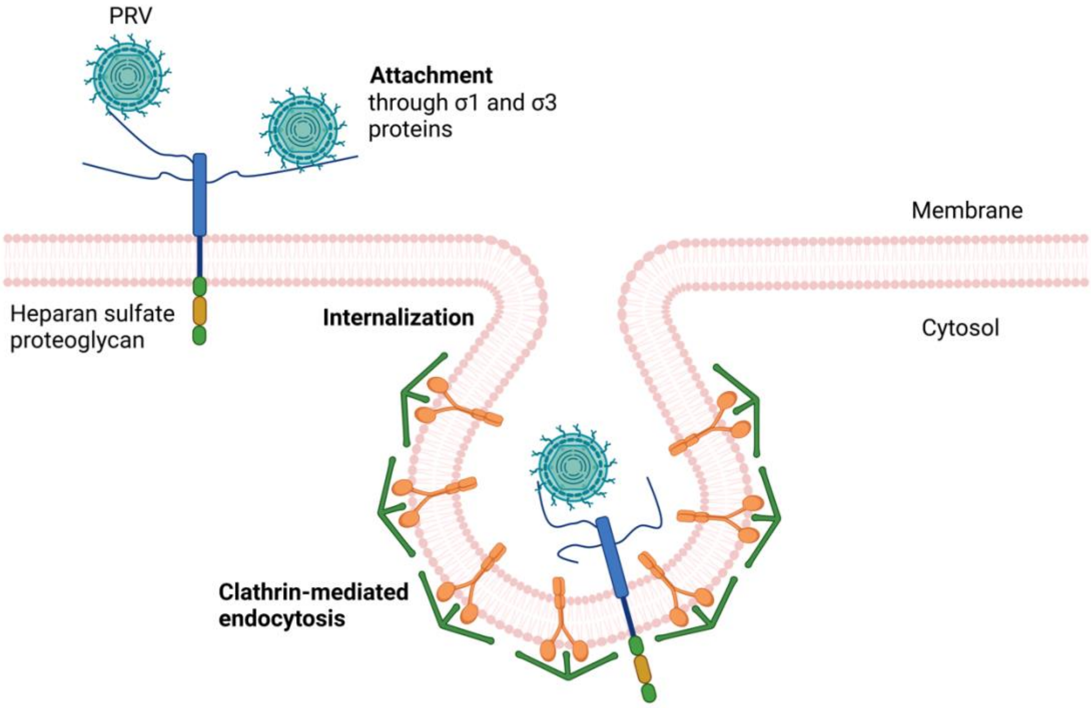

Supplementary Fig 3. Graphical illustration of potential mechanism underlying HS-dependent PRV entry. Binding of PRV to HS chain on proteoglycan could activate the internalization of PRV through clathrin-mediated endocytosis.
